# Supplementary material for: An Enantioselective Approach to 4-Substituted Proline Scaffolds: Synthesis of (S)-5-(tert-Butoxy carbonyl)-5-azaspiro[2.4]heptane-6-carboxylic Acid
Source: Molecules. 2020 Nov 30;25(23):5644. doi: 10.3390/molecules25235644 (PMC7729483; doi:10.3390/molecules25235644)
Supplement: Supplementary file 1 [file molecules-25-05644-s001.pdf]

SUPPLEMENTARY MATERIALS

An Enantioselective Approach to 4-Substituted Proline Scaffolds: Synthesis of (*S*)-5-(*tert*-butoxycarbonyl)-5-azaspiro[2.4]heptane-6-carboxylic acid

Blanca López [1,2], Martí Bartra [2], Ramon Berenguer [2], Xavier Ariza [1,3,4,*], Jordi Garcia [1,3,4,*], Roberto Gómez [1,3,4], and Hèctor Torralvo [1,2]

[1]Departament de Química Inorgànica i Orgànica, Secció de Química Orgànica, Facultat de Química, Universitat de Barcelona, Martí i Franquès 1-11. 08028 Barcelona, Spain.

[2]R&D Department, Esteve Química S.A., Caracas 17-19, 08030 Barcelona, Spain.

[3]Institut de Biomedicina (IBUB), Universitat de Barcelona, Barcelona, Spain.

[4]CIBER Fisiopatología de la Obesidad y la Nutrición (CIBERobn), Instituto de Salud Carlos III, Madrid, Spain.

E-mail: jordigarciagomez@ub.edu, xariza@ub.edu

TABLE OF CONTENTS:

^{1}H NMR and ^{13}C NMR spectra of **1** 2

^{1}H NMR and ^{13}C NMR spectra of **5** 3

^{1}H NMR and ^{13}C NMR spectra of **6** 4

^{1}H NMR and ^{13}C NMR spectra of **12** 5

^{1}H NMR and ^{13}C NMR spectra of **13** 6

^{1}H NMR and ^{13}C NMR spectra of **14** 7

^{1}H NMR and ^{13}C NMR spectra of benzyl ester of **1** 8

HPLC chromatograms of compound **5** benzyl ester 9

HPLC chromatograms of compound **12** Cbz derivative 10

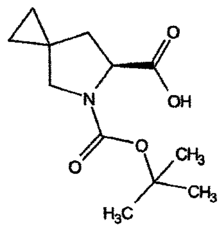

Compound **1**
^{1}H NMR (400 MHz, $CDCl_3$)

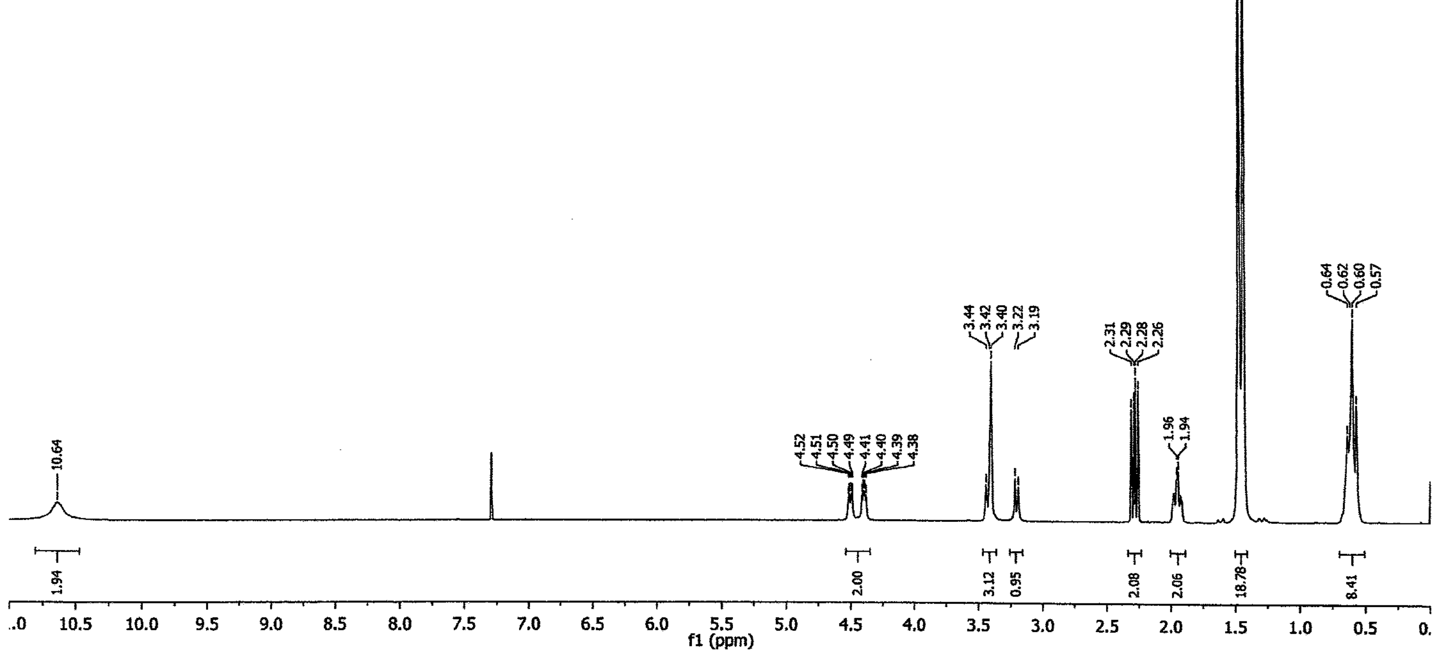

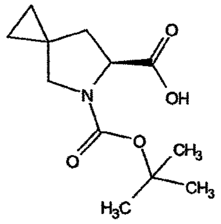

Compound **1**
^{13}C NMR (101 MHz, $CDCl_3$)

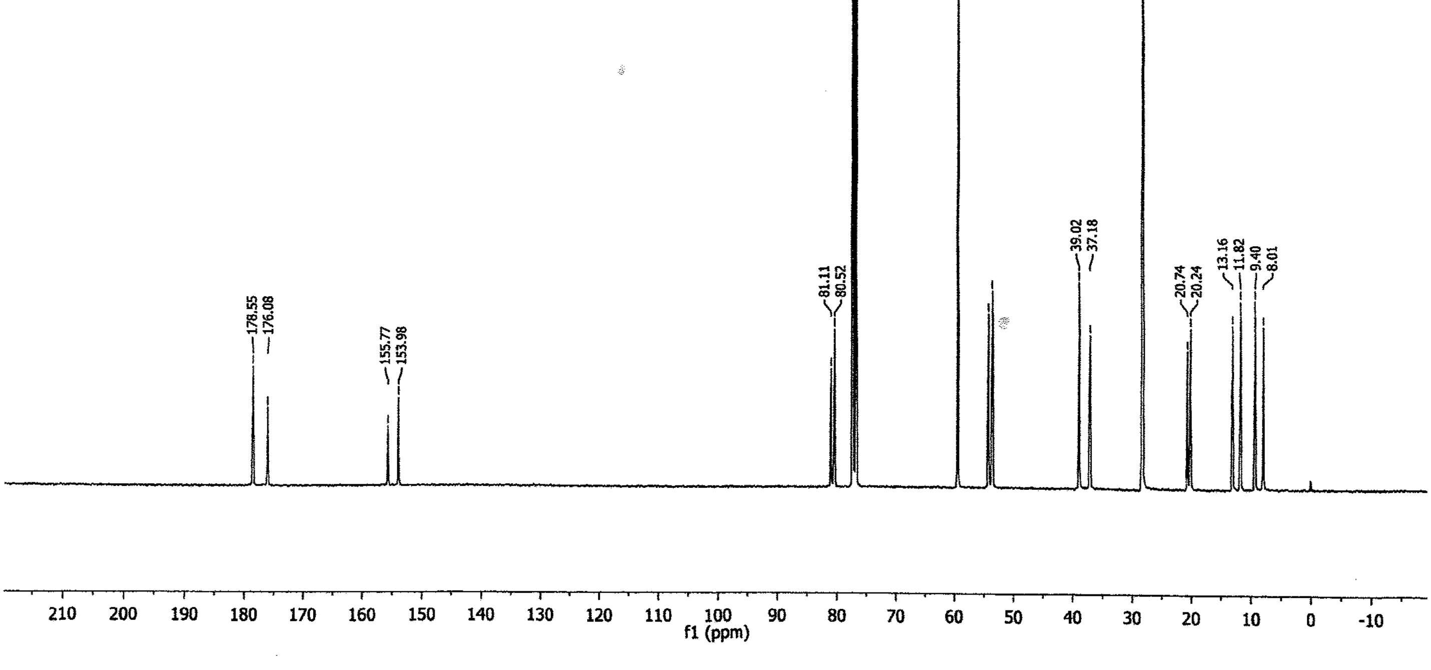

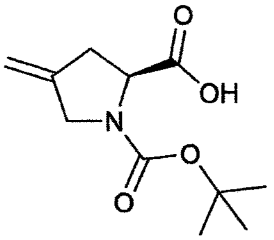

Compound **5**
^{1}H NMR (400 MHz, $CDCl_3$)

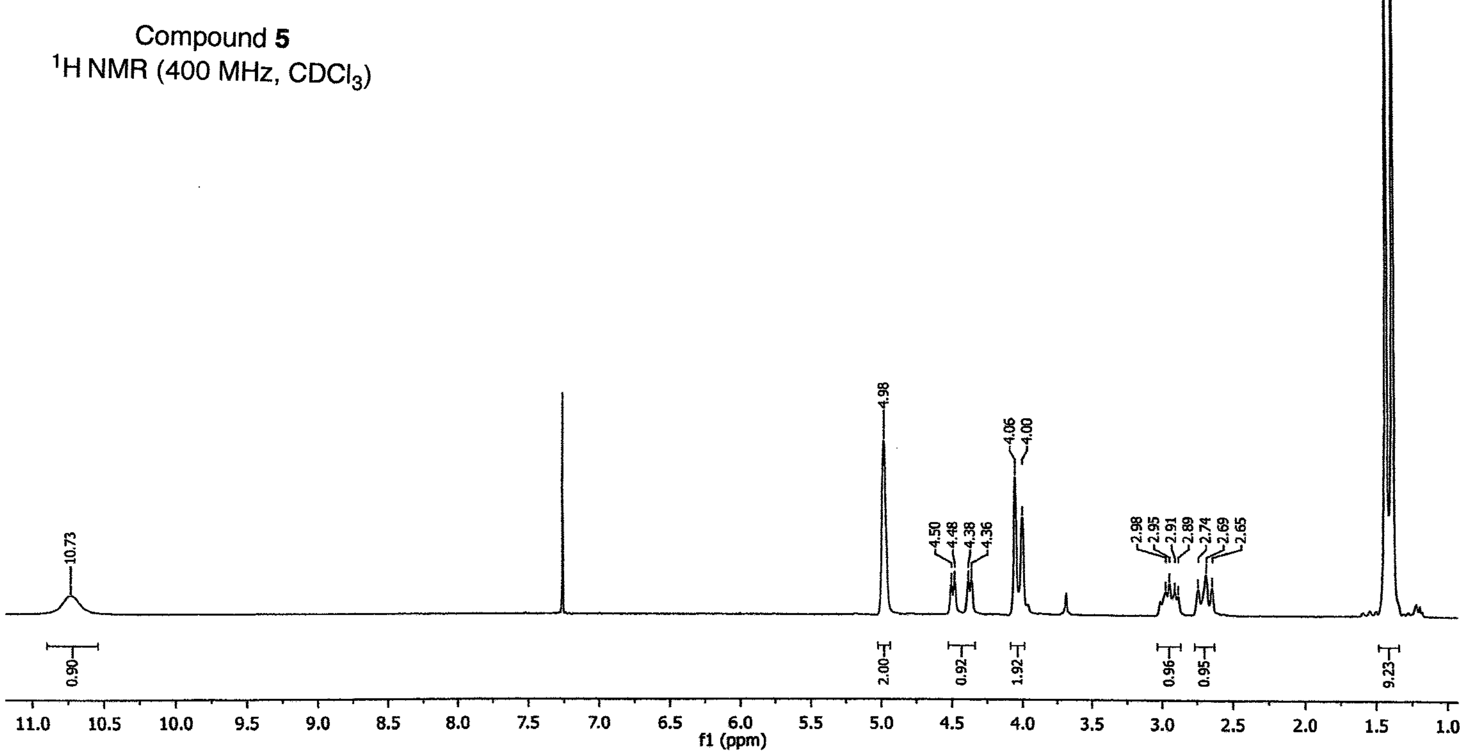

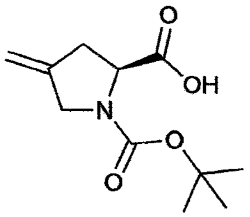

Compound **5**
^{13}C NMR (101 MHz, $CDCl_3$)

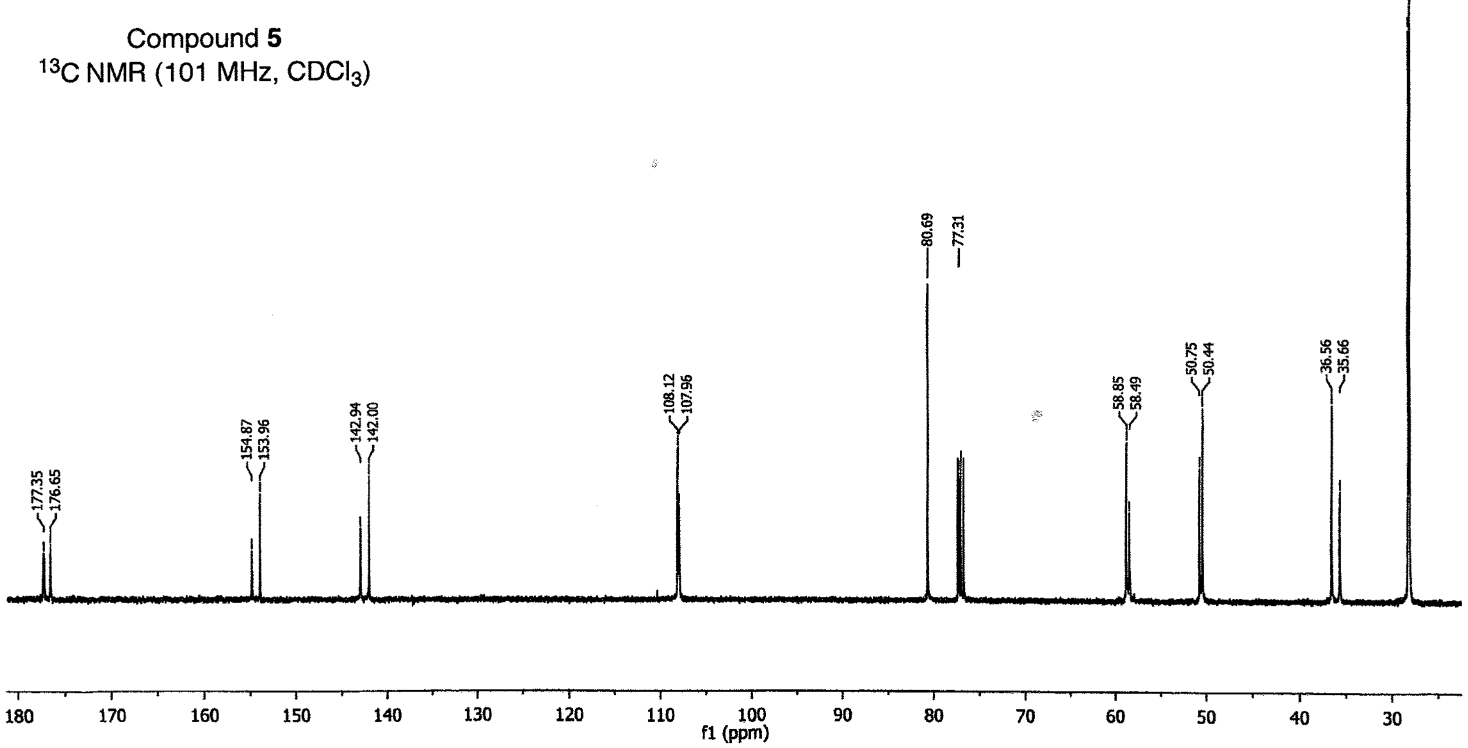

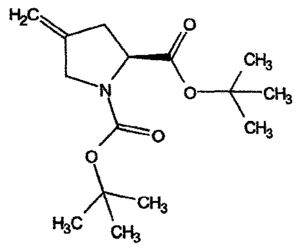

Compound **6**
^{1}H NMR (400 MHz, $CDCl_3$)

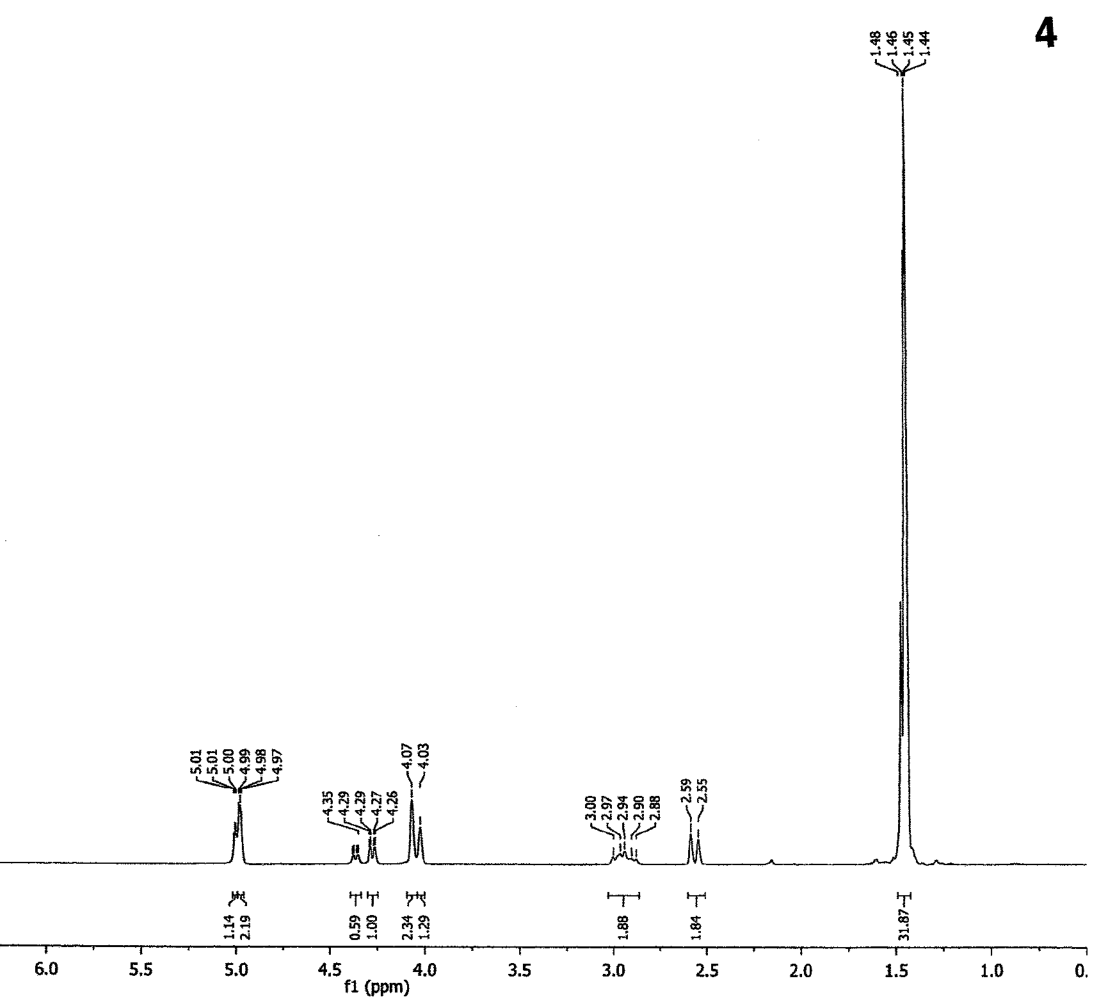

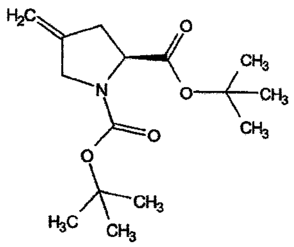

Compound **6**
^{13}C NMR (101 MHz, $CDCl_3$)

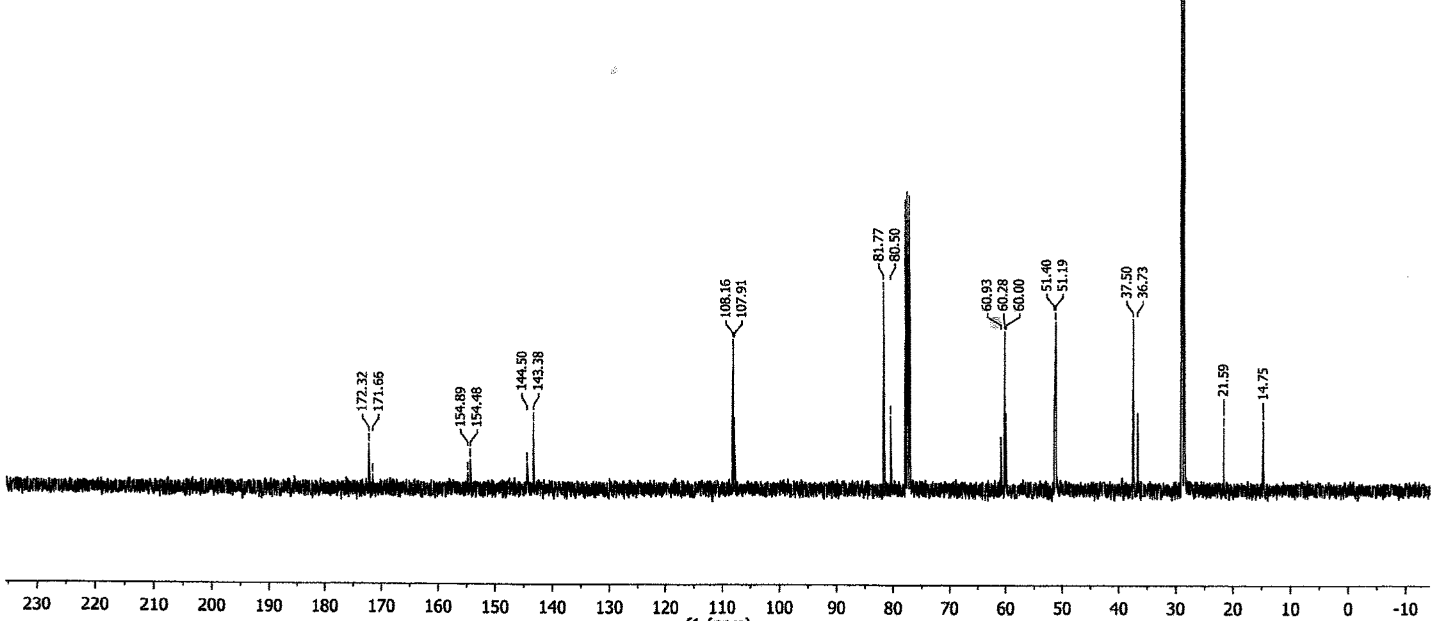

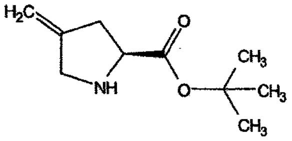

Compound **12**
^{1}H NMR (400 MHz, $CDCl_3$)

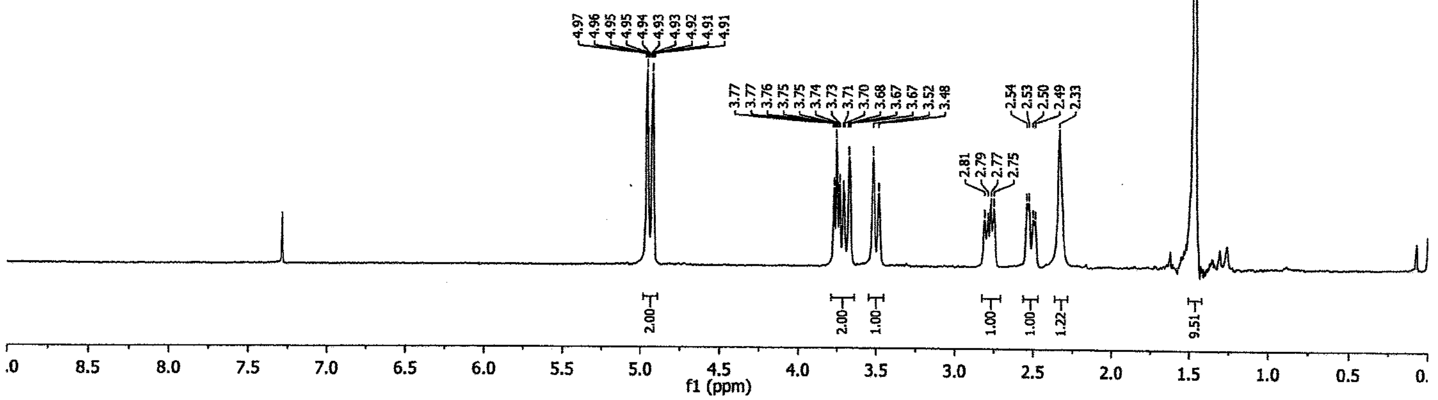

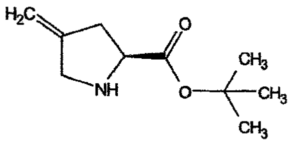

Compound **12**
^{13}C NMR (101 MHz, $CDCl_3$)

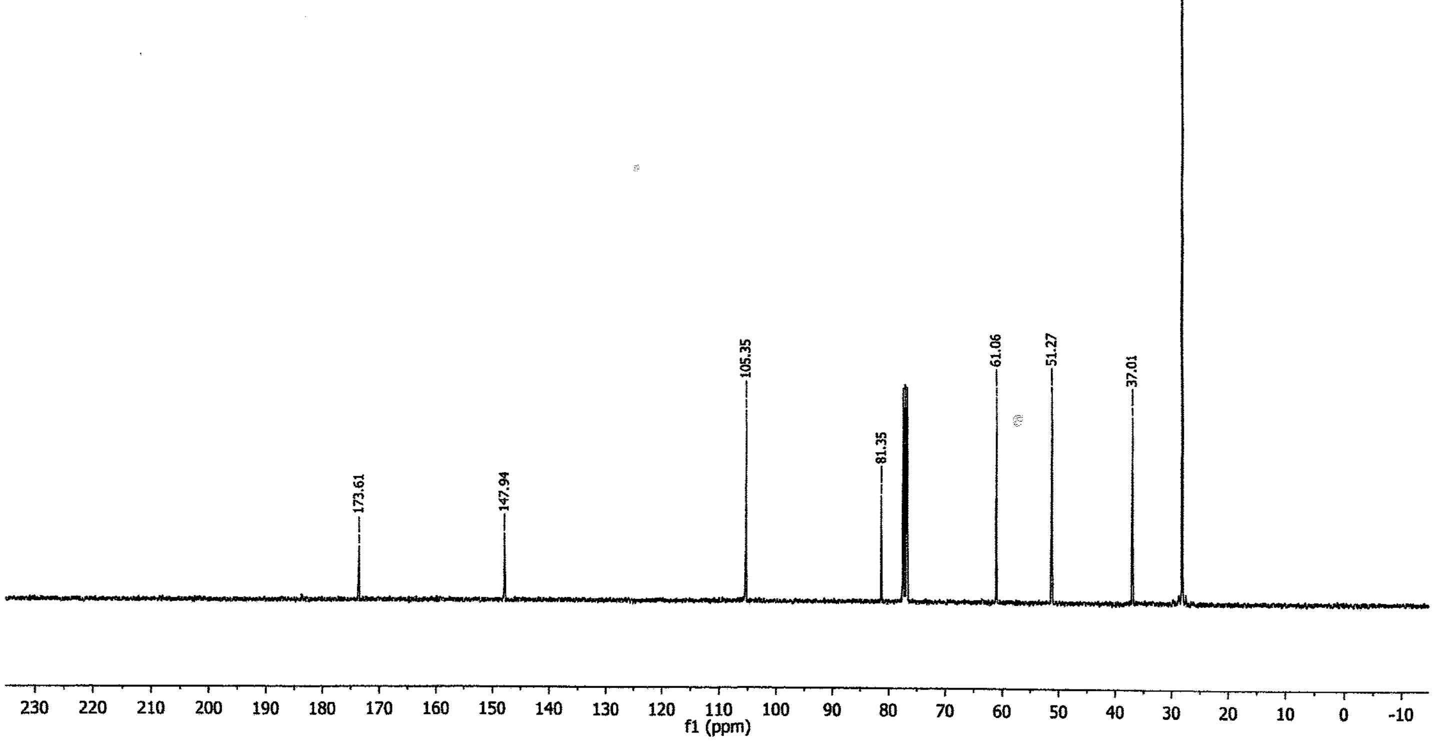

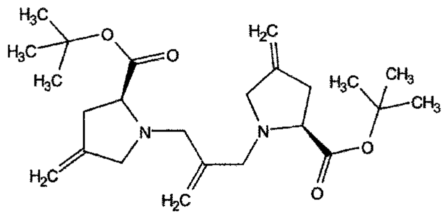

Compound **13**
^{1}H NMR (400 MHz, $CDCl_3$)

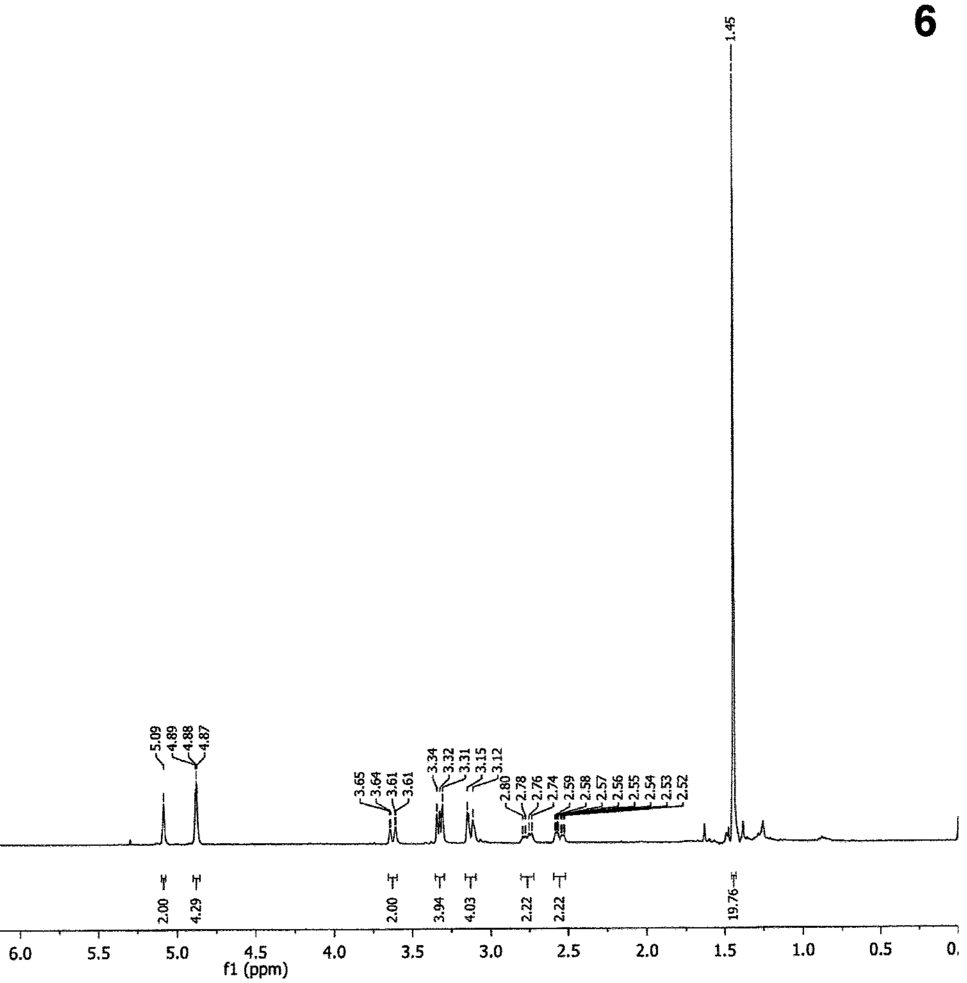

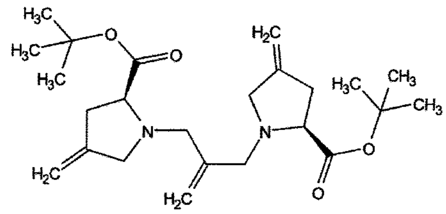

Compound **13**
^{13}C NMR (101 MHz, $CDCl_3$)

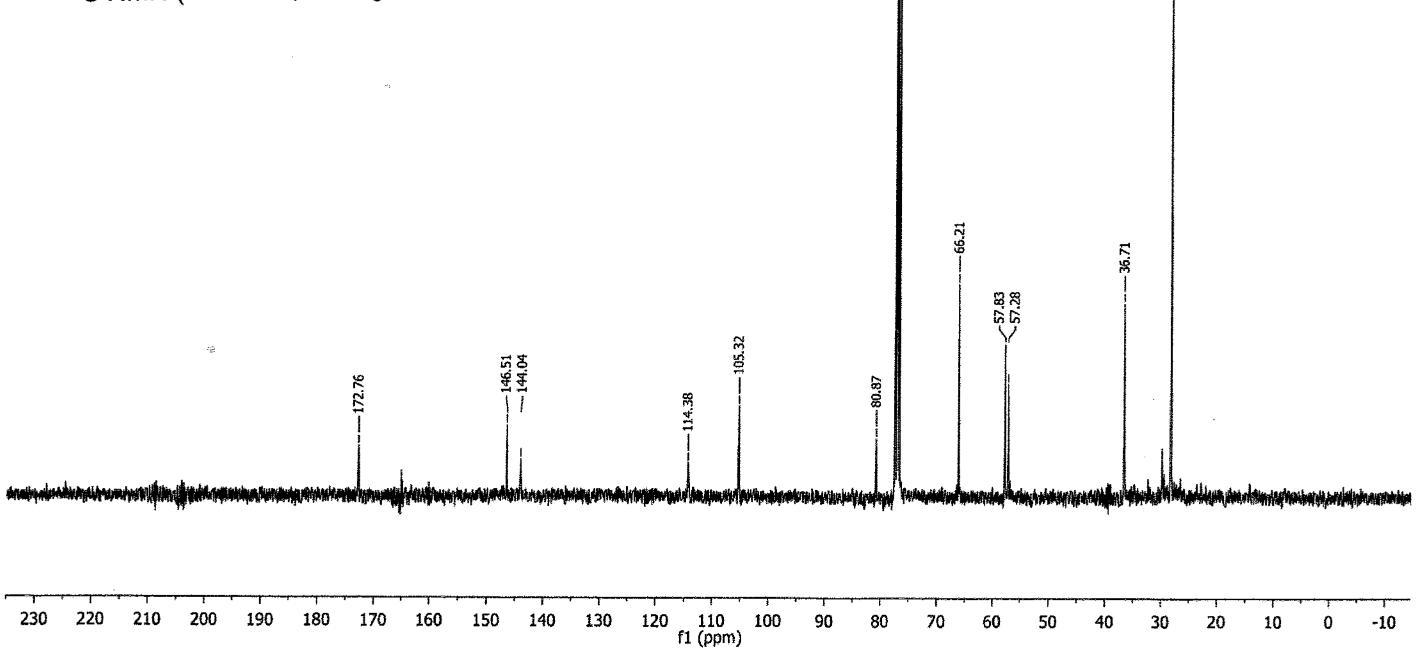

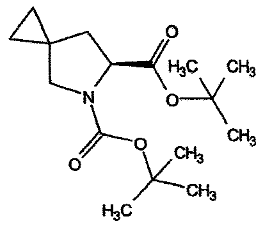

Compound **14**
^{1}H NMR (400 MHz, $CDCl_3$)

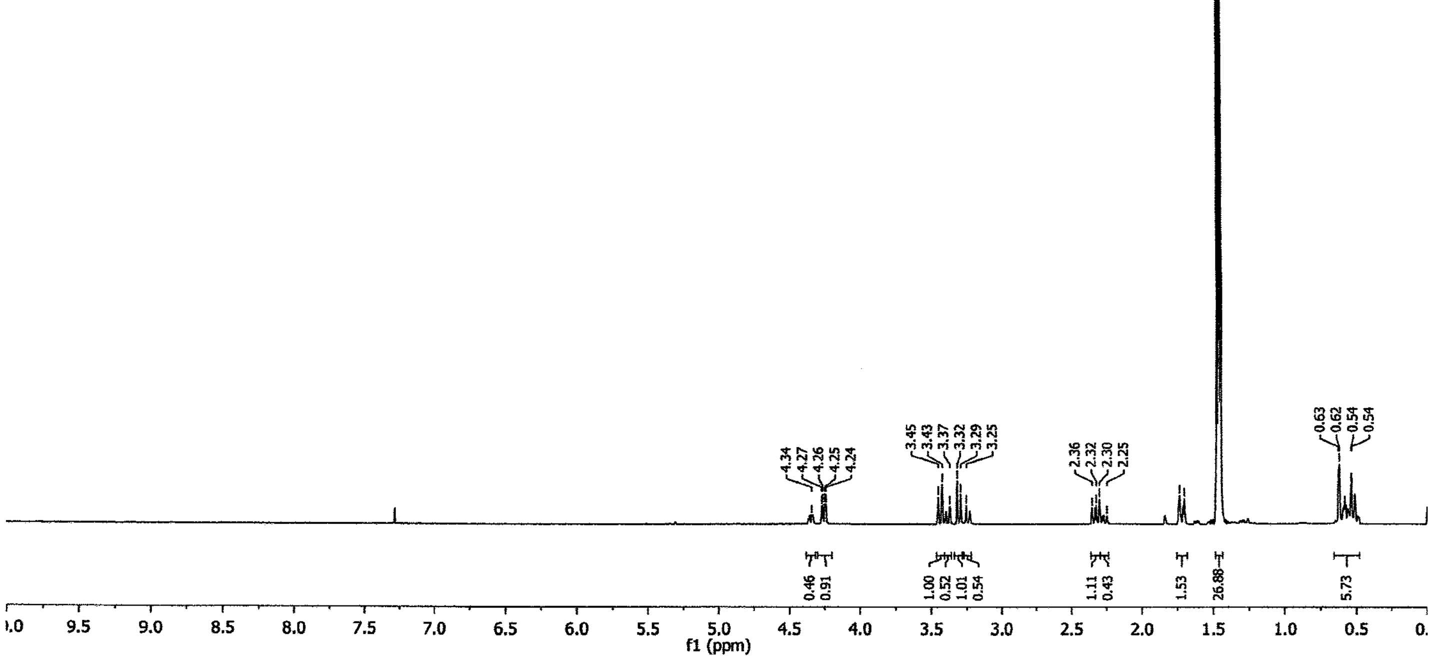

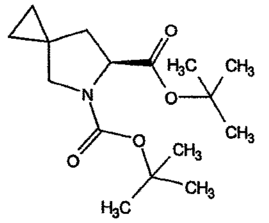

Compound **14**
^{13}C NMR (101 MHz, $CDCl_3$)

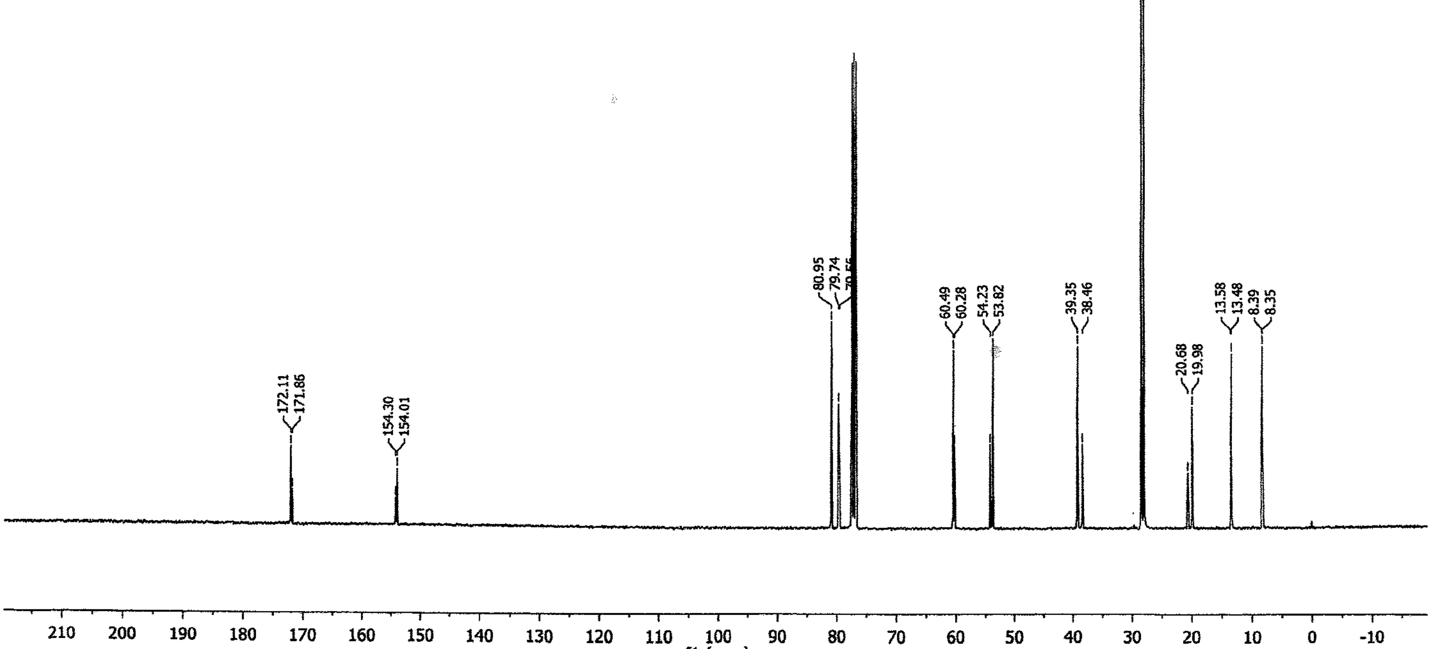

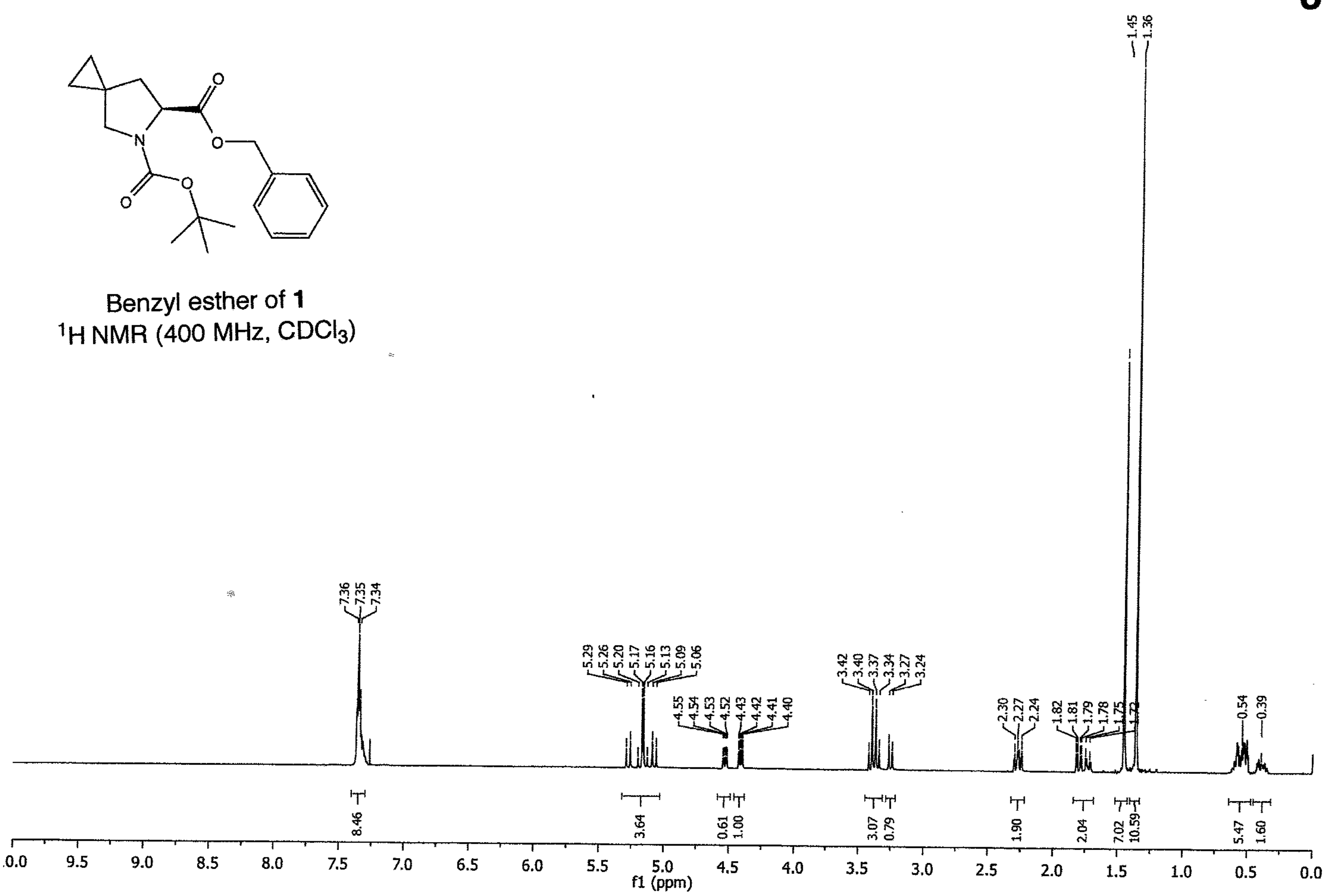

Benzyl esther of **1**
^{1}H NMR (400 MHz, $CDCl_3$)

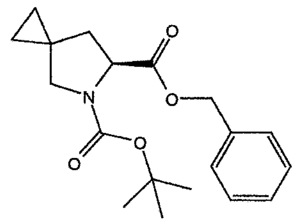

Benzyl esther of **1**
^{13}C NMR (101 MHz, $CDCl_3$)

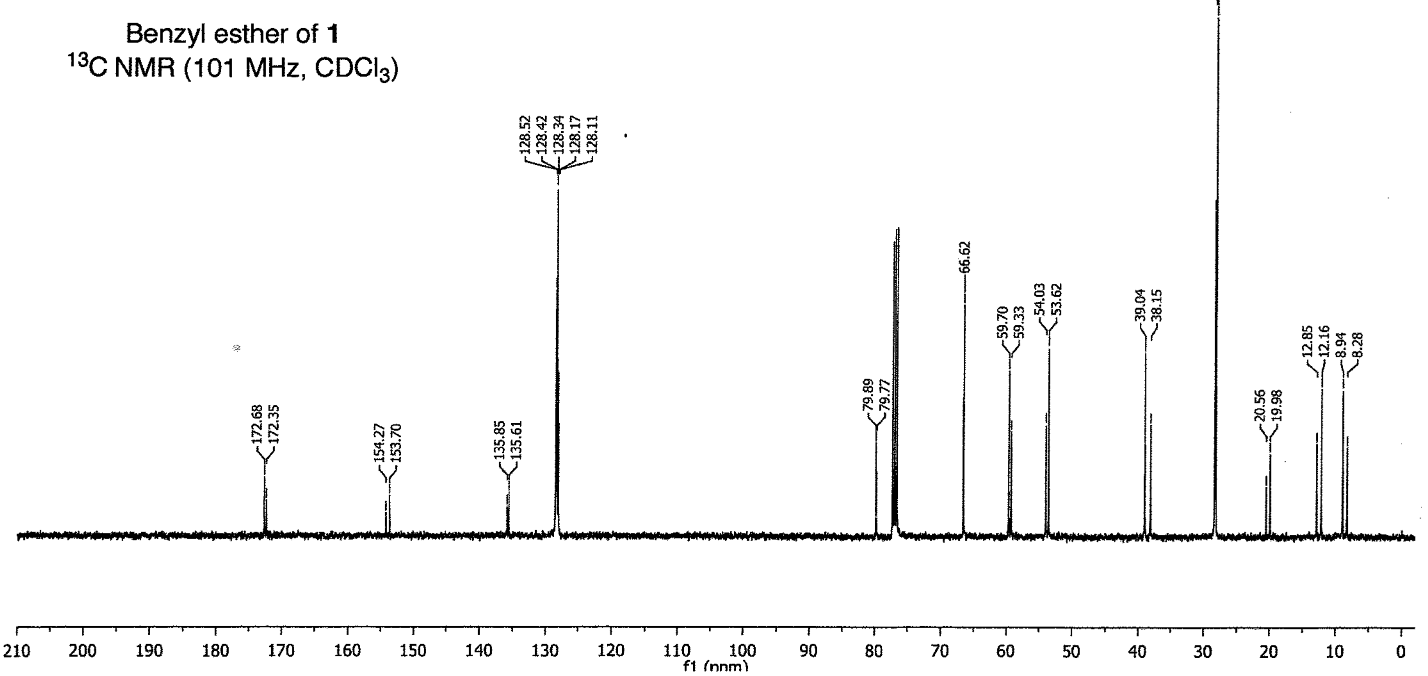

HPLC chromatograms with a CHIRALPACK® IC (0.46 cm x 25 cm) column using a 92:8 hexanes/isopropanol mixture as eluent of benzyl ester of **5**

A) Racemic mixture

B) 95:5 enantioenriched sample

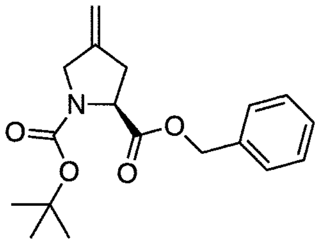

Benzyl ester of **5**
(*S*)-isomer

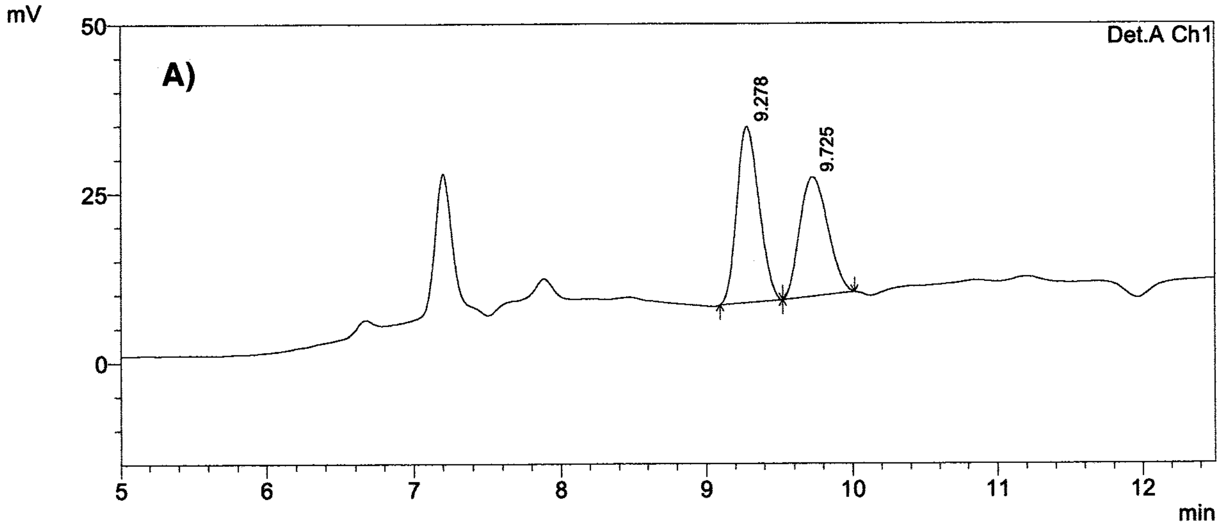

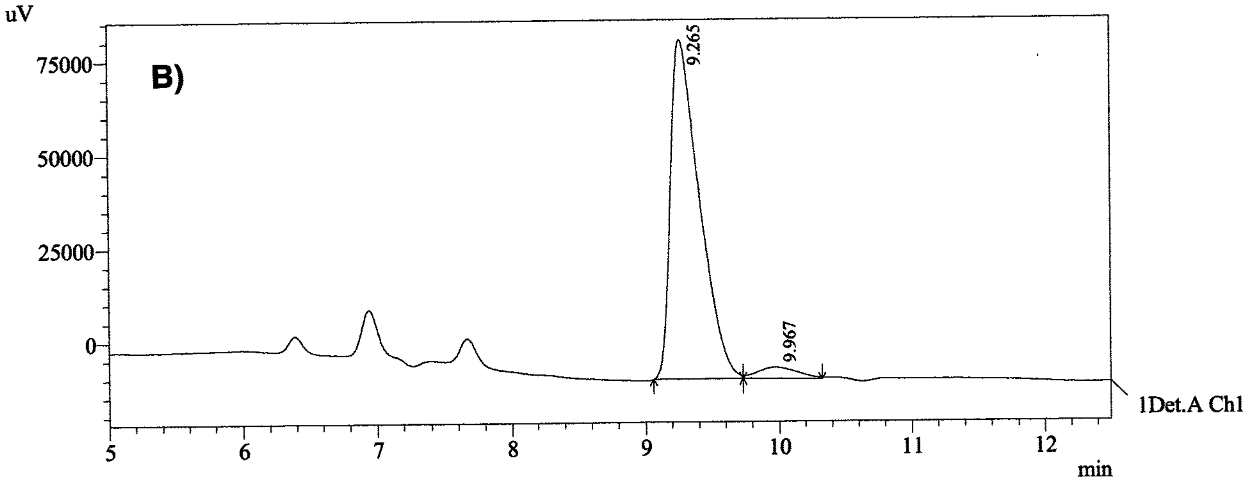

1 Det.A Ch1 / 254nm

PeakTable

Detector A Ch1 254nm

Peak#	Ret. Time	Area	Height	Area %	Height %
1	9.265	1367111	90553	95.489	96.735
2	9.967	64587	3056	4.511	3.265
Total		1431698	93609	100.000	100.000

HPLC chromatograms of *N*-Cbz derivative of **12** with a CHIRALPACK® IA (0.46 cm x 25 cm) column using a 90:10 hexanes/isopropanol mixture as eluent. **10**

A) Racemic mixture

B) 95:5 enantioenriched sample

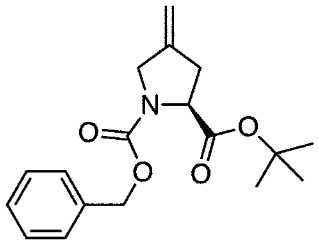

Cbz derivative of **12**
(*S*)-isomer

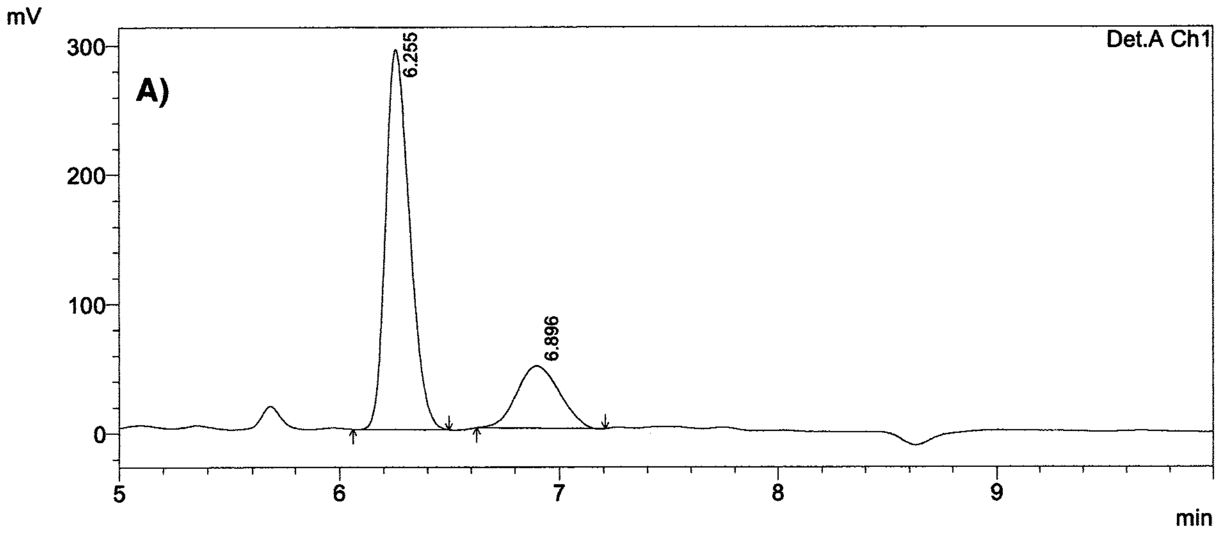

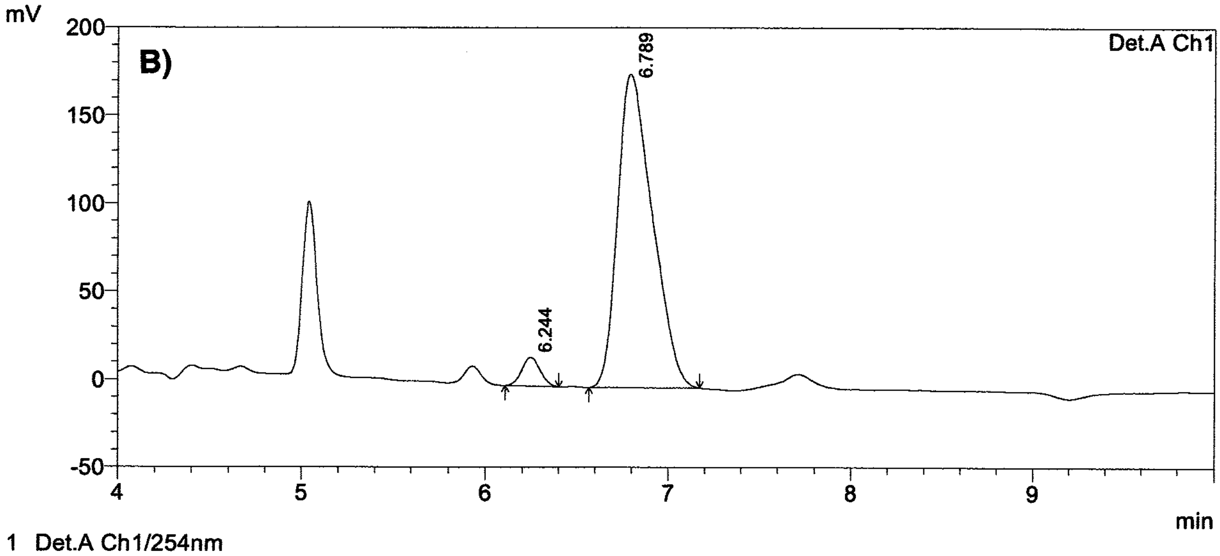

1 Det.A Ch1/254nm

PeakTable

Detector A Ch1 254nm

Peak#	Ret. Time	Area	Height	Area %	Height %
1	6.244	114085	16233	4.628	8.356
2	6.789	2351212	178047	95.372	91.644
Total		2465296	194280	100.000	100.000
